# Aging Increases Hypoxia-Induced Endothelial Permeability and Blood-Brain Barrier Dysfunction by Upregulating Arginase-II

**DOI:** 10.14336/AD.2023.1225

**Published:** 2024-01-17

**Authors:** Xin Cheng, Duilio M. Potenza, Andrea Brenna, Guillaume Ajalbert, Zhihong Yang, Xiu-Fen Ming

**Affiliations:** Laboratory of Cardiovascular and Aging Research, Department of Endocrinology, Metabolism, and Cardiovascular System, Faculty of Science and Medicine, University of Fribourg, Switzerland

**Keywords:** aging, arginase-II (Arg-II), blood-brain barrier (BBB), endothelial cell-cell junctions, hypoxia, mitochondrial reactive oxygen species (mtROS)

## Abstract

Increased endothelial permeability plays an important role in blood-brain barrier (BBB) dysfunction and is implicated in neuronal injury in many diseased conditions. BBB disruption is primarily determined by dysfunction of endothelial cell-cell junctions. Deprivation of oxygen supply or hypoxia, a common feature of a variety of human diseases, is a major risk factor for BBB disruption. The molecular regulatory mechanisms of hypoxia-induced BBB dysfunction remain incompletely understood. The mitochondrial enzyme, arginase type II (Arg-II), has been shown to promote endothelial dysfunction. However, its role in hypoxia-induced BBB dysfunction has not been explored. In the C57BL/6J mouse model, hypoxia (8% O_2_, 24 hours) augments vascular Arg-II in the hippocampus, decreases cell-cell junction protein levels of Zonula occludens-1 (ZO-1), occludin, and CD31 in endothelial cells, increases BBB leakage in the brain in old mice (20 to 24 months) but not in young animals (3 to 6 months). These effects of hypoxia in aging are suppressed in *arg-ii^-/-^* mice. Moreover, the age-associated vulnerability of endothelial integrity to hypoxia is demonstrated in senescent human brain microvascular endothelial cell (hCMEC/D3) culture model. Further results in the cell culture model show that hypoxia augments Arg-II, decreases ZO-1 and occludin levels, and increases endothelial permeability, which is prevented by *arg-ii* gene silencing or by inhibition of mitochondrial reactive oxygen species (mtROS) production. Our study demonstrates an essential role of Arg-II in increased endothelial permeability and BBB dysfunction by promoting mtROS generation, resulting in decreased endothelial cell-cell junction protein levels under hypoxic conditions particularly in aging.

## INTRODUCTION

Blood-brain barrier (BBB) protects central nervous system (CNS) from potentially harmful components in circulating blood and allows nutrient transport for CNS [1]. The properties of BBB are primarily determined by brain capillary endothelial cells which do not have fenestrations, exhibit low pinocytotic activities, and possess tight cell-cell junctions including tight junctions (TJ) and adherens junctions (AJ) [2]. Disruption of BBB integrity due to destabilization of endothelial cell-cell junctional contacts occurs in many pathological conditions including inflammation, brain tumor, ischemic stroke and hypoxia, etc., which leads to exacerbation of neuronal injury and eventually death [2, 3]. Hypoxia is a common feature of a broad spectrum of human diseases including obstructive sleep apnea, chronic obstructive pulmonary disease (COPD), pulmonary hypertension, cardiac and cerebrovascular disorders [4]. All of these situations are highly associated with aging [4]. Hypoxia is known to induce BBB breakdown, leading to neurological disorders [5, 6]. Among the endothelial cell-cell junctional proteins, the TJ protein Zonula occludens-1 (ZO-1) and occludin as well as the AJ protein VE-cadherin are arguable of dominant importance [7]. Consistent with this notion, evidence has been presented that hypoxia-induced hyperpermeability involves decreased occludin and VE-cadherin levels during an ischemic stroke and OSA, leading to the disruption of BBB [8, 9]. At the molecular level, reactive oxygen species (ROS) has been demonstrated to be a potential mechanism leading to alterations of the endothelial junctions and BBB dysfunction [10, 11]. Despite extensive research on the cerebrovascular endothelial cell-cell junctions and BBB disruption, studies addressing the underlying molecular mechanisms are currently very limited. Much research is needed to better understand the molecular mechanisms governing BBB integrity and to find novel therapeutic targets in various clinical settings.

The hippocampus, particularly the CA1 area is highly vascularized and seems more sensitive to hypoxia and ischemia than other areas [12]. Recent studies provide compelling evidence demonstrating that BBB integrity deteriorates with aging [14, 15] and becomes more vulnerable to stressors such as hypoxia [1, 16]. This age-associated increase in BBB sensitivity to stressors is suggested to play an important role in neurodegenerative diseases [1, 2, 17]. The mechanism of BBB disruption, particularly, the age-associated susceptibility of BBB to stressors such as hypoxia, remains elusive. Therefore, further exploration of cellular and molecular mechanisms of BBB disruption would lead to the discovery of novel strategies to prevent BBB dysfunction and shall help to minimize neuronal injury in various pathological conditions.

In the past, arginase, an enzyme metabolizing L-arginine to L-ornithine and urea, has been implicated in endothelial dysfunction and aging [18, 19]. There are two isoforms, namely arginase type I (Arg-I) and arginase type II (Arg-II) [20]. Arg-I is mainly expressed in hepatocytes and Arg-II in the kidney. Arg-I is located in the cytosol, whereas Arg-II is in the mitochondria [18, 20]. Notably, Arg-II is the main isoform that is inducible in human endothelial cells [18, 19]. Accumulating studies indicate an important role of Arg-II in cardiovascular diseases, cellular senescence, and organism aging accompanied by enhanced ROS production [19, 21]. However, it remains elusive whether and how Arg-II regulates endothelial cell-cell junctions linking to hypoxia-induced BBB disruption. Studies show that Arg-II is indeed upregulated in response to hypoxia in endothelial cells, which contributes to the hypoxia-induced endothelial dysfunction and mitochondrial ROS (mtROS) production [22].

Given the important role of Arg-II in endothelial dysfunction and hypoxia-induced endothelial ROS production and inflammation [22], this study aims to investigate whether and how Arg-II regulates endothelial cell-cell junctions in hypoxia-induced cerebrovascular endothelial hyperpermeability and BBB disruption, and whether Arg-II plays a role in age-associated BBB vulnerability in response to acute hypoxia.

## MATERIALS AND METHODS

### Reagents and materials

Reagents were purchased from the following sources: EBM-2 endothelial basal medium (#cc-3156) was obtained from Lonza (Basel, Switzerland). Ascorbic acid (#A4544), human Basic Fibroblast Growth Factor (#F0291) and hydrocortisone (#H0888) were obtained from Sigma-Aldrich. Chemically defined Lipid Concentrate (#11905031) and HEPES (#15630-080) were obtained from Life Technologies. M.O.M (mouse on mouse) blocking reagent (MKB-2213) was obtained from Vector Laboratories. QuickTiter™ Adenovirus Titer Immunoassay Kit (VPK-109, Cell Biolabs, Inc) were purchased from LuBioScience GmbH (Luzern, Switzerland). The manufacturer catalog numbers and dilution of all antibodies used for Western Blot (WB) and immunofluorescence (IF) staining are listed below: rabbit antibody against Arg-II (#55003, WB 1:1000; IF 1:100), p-p53 (#9284s, WB 1:1000; IF 1:100), p21 (#2947s, WB 1:1000; IF 1:100) and IgG (#3900s, IF 1:100) were obtained from Cell Signaling Technology (Danvers, USA). Rabbit antibodies against ZO-1 (ab216880, WB 1:1000; IF 1:100), occludin (ab235986, WB 1:1000; IF 1:100) and VE-cadherin (ab33168, IF 1:100) were obtained from Abcam. Goat antibody against CD31 (sc-1506, IF 1:50) and mouse antibody against IgG (sc-2025, IF 1:1000) were obtained from Santa Cruz Biotechnology. Rat antibody against CD31 (REF14-0311-82, IF 1:100) and Alexa fluor 680-conjugated goat anti-mouse IgG (A-21057, WB 1:5000) were purchased from Invitrogen (Lucerne, Switzerland). Mouse-antibody against vinculin (MCA465GA, WB 1:10’000) was obtained from Bio-Rad. Mouse antibody against GAPDH (10R-G109A, WB 1:10’000) and β-actin (A5441, WB 1:10’000) were purchased from Sigma-Aldrich (Buchs, Switzerland). IRDye 800-conjugated affinity purified goat anti-rabbit IgG (9263221, WB 1:5000) was from BioConcept (Allschwil, Switzerland). Alexa Fluor 488-conjugated goat anti-mouse IgG (H+L) secondary Ab (A-11001, IF 1:400), Alexa Fluor 488-conjugated goat anti-rabbit IgG (H+L) secondary Ab (A-11008, IF 1:400), Alexa Fluor 488-conjugated donkey anti-goat IgG (H+L) secondary Ab (A-11055, IF 1:400), Alexa Fluor 488-conjugated goat anti-rat IgG (H+L) secondary Ab (A-11006, IF 1:400), Fluor 568-conjugated goat anti-mouse IgG (H+L) secondary Ab (A-11031, IF 1:400), Alexa Fluor 594-conjugated goat anti-rabbit IgG (H+L) secondary Ab (A-11072, IF 1:400) were purchased from Thermo Fisher Scientific (Waltham, Massachusetts, USA).

### Animals and hypoxia experiments

*Arg-ii* gene knockout (*arg-ii^-/-^*) mice were kindly provided by Dr. William O’Brien [23] and backcrossed to wild type (*wt*) C57BL/6J mice for more than 10 generations. Genotypes of mice were confirmed by polymerase chain reaction (PCR) as previously described [19]. Offspring of *wt* and *arg-ii^-/-^* mice was generated by interbred from hetero/hetero cross. Mice were housed at 23°C with a 12h light-dark cycle. Animals were fed a normal chow diet and had free access to water. For hypoxia experiments, young (3-6 months in age) and old (20-24 months in age) male *wt* and *arg-ii^-/-^* mice were randomly allocated into two groups that were continuously exposed to normoxia (21% O_2_) or hypoxia (8% O_2_) for 24 hours in the control or hypoxia cabinet, respectively, of a Coy *In Vivo* Hypoxic Cabinet System (The Coy Laboratory Products, Grass Lake, MI, United States). 24 hours after continuous exposure to either normoxia or hypoxia, mice were euthanized under deep anesthesia (i.p. injection of a mixture of ketamine/xylazine 50 mg/kg and 5 mg/kg, respectively) and death was confirmed by absence of all the reflexes and by exsanguination. Brains were perfused with 0.9% NaCl and 4% PFA. Perfused brains were cryoprotected by 30% sucrose solution and sectioned (40 mm, coronal) using a cryostat for immunofluorescence staining experiments. Experimental work with animals was approved by the Ethical Committee of the Veterinary Office of Fribourg Switzerland (2020-01-FR) and performed in compliance with guidelines on animal experimentation at our institution.

### Recombinant Adenovirus (rAd)

rAd expressing *lacz* or *arg-ii* driven by CMV promoter (rAd/CMV-*lacz* or -*arg-ii*) and shRNA targeting *lacz* or human *arg-ii* driven by U6 promoter (rAd/U6-*lacz*^shRNA^ or -h*arg-ii*^shRNA^) were generated with the Gateway Technology (Invitrogen life Technologies) according to manufacturer’s instruction [24]. rAd/CMV-*lacz* and rAd/U6-*lacz*^shRNA^ were used as control for rAd/CMV-*arg-ii* and rAd/U6-h*arg-ii*^shRNA^, respectively. The pCMV6 construct encoding the murine *arg-ii* was obtained from Origene. The human *arg-ii* targeting sequence for rAd/U6-h*arg-ii*^shRNA^ is indicated in boldface and underlined below (only the sense strand is shown): CAC CGCGAGTGCATTCCATCCTGAACGAATTCAGGATGGAATGCACTCGC.

The rAd titer (infectious unit: ifu/ml) was determined by staining the largest and most abundant structural proteins “hexon proteins” in the adenovirus capsid using QuickTiter™ Adenovirus Titer Immunoassay Kit after infecting HER911 cells, derived from human retina cells by Adenovirus E1 transformation for 48 hours [25].

### Endothelial cell culture and adenoviral transduction

hCMEC/D3 (human brain microvascular endothelial cell line) cells were cultured in EBM-2 Medium containing 5% fetal bovine serum (FBS), 1.4 µmol/L hydrocortisone, 5 µg/mL acid ascorbic, 1/100 chemically defined lipid concentrate, 10 mmol/L HEPES, 1 ng/mL bFGF (human basic fibroblast growth factor) and 100 U/mL penicillin, 100 μg/mL streptomycin. The cells were maintained at 37°C in a humidified incubator containing a 5% CO_2_ atmosphere. To silence *arg-ii*, the cells were seeded in 6-cm^2^ dish for 24 h and transduced with (rAd)/U6-*lacz*^shRNA^ as control or rAd/U6-*arg-ii*^shRNA^ at titers of 100 multiplicity of infection (MOI) and cultured in complete medium for two days before experiments.

To overexpress *arg-ii*, the cells were seeded in 6-cm^2^ dish for 24 h and transduced with rAd/CMV-*lacz* as control or rAd/CMV-*arg-ii* for 48 h at the titers of 50-100 MOI and cultured in complete medium for two days. The cells were then either harvested for WB or subjected to analysis of mtROS or permeability assay.

### Induction of endothelial cell senescence

hCMEC/D3 cells were treated with 100 µmol/L of H_2_O_2_ for 12 to 24 hours to induce premature senescence and then used for further experiments.

### Hypoxia experiments with cultured endothelial cells

For hypoxia experiments, hypoxic conditions were achieved by placing the cultures dishes or plates in a Coy *In Vitro* Hypoxic Cabinet System (The Coy Laboratory Products, Grass Lake, MI USA) with 5% CO_2_ and N_2_ as balance [22]. Non-senescent and senescent cells were incubated at 6% O_2_ for the senescence-associated vulnerability of endothelial permeability in response to hypoxia. For studying the underlying mechanisms of hypoxia-induced increase in endothelial permeability, non-senescent “Young” cells, and more severe acute hypoxia, i.e., 1% O_2_ were used.

### Western Blot (WB)

Cell lysate preparation, SDS-PAGE and WB, antibody incubation, and signal detection were performed as described previously [10]. GAPDH and vinculin were used as protein loading controls. Briefly, cell extracts were prepared by lysing cells on ice for 15 minutes in the ice-cold lysis buffer with the following composition (mmol/L): 20 Tris.HCl, 138 NaCl, 2.7 KCl with pH 8.0, 1 MgCl_2_, 1 CaCl_2_, 1 sodium-o-vanadate, 0.02 leupeptin, 0.018 pepstatin, 5 EDTA and 20 NaF supplemented with 5% glycerol, 1% NP-40. Cell debris and nuclei were removed by centrifugation (Sorvall Legend Micro 17R) at 12,000 × *g* for 15 minutes at 4°C. Protein concentrations of the supernatant were then determined by the Lowry method (500-0116, Bio-Rad). An equal amount of protein from each sample was heated at 95°C for 5 minutes in a loading buffer and separated by SDS-PAGE electrophoresis. Proteins in the SDS-PAGE gel were then transferred to PVDF membranes which were blocked with PBS-Tween-20 supplemented with 5% skimmed milk. The membranes were then incubated with the corresponding primary antibody overnight at 4°C with gentle agitation. After washing with blocking buffer, the membranes were then incubated with corresponding anti-mouse (Alexa Fluor 680-conjugated) or anti-rabbit (IRDye 800-conjugated) secondary antibodies. Signals were visualized using the Odyssey Infrared Imaging System (LI-COR Biosciences) and quantified by NIH Image J 1.60 (US NIH).

### Mitochondrial superoxide detection (MitoSOX staining) in cultured cells

Mitochondrial superoxide generation was monitored by using MitoSOX [22]. Briefly, the cells were incubated with MitoSOX at the concentration of 5 μmol/L for 10 minutes. After washing, the cells were then fixed with 3.7% PFA followed by counterstaining with Hoechst 33342 and then subjected to imaging through 40× objectives with Leica TCS SP5 confocal laser microscope. To study the role of mtROS in the regulation of cell-cell junctional proteins, cells were treated with rotenone (2 μmol/L, 1 hour) followed by MitoSOX staining and WB or immunostaining analysis of cell-cell junctional proteins as described above.

### Immunofluorescence staining

Immunofluorescence staining was performed as described previously with minor modifications [26]. The secondary antibody-only controls were employed for validation of the specific signals from the relevant primary antibodies. For anti-Arg-II, tissues from *arg-ii*^-/-^ mice were also employed for antibody validation. Briefly, mice were perfused with 0.9% NaCl followed by 4% PFA. Subsequently, the brains were isolated, kept in 4% PFA for 15-20 hours, and then cryoprotected with 30% sucrose solution and sectioned using a cryostat. Coronal sections (40 µm) of the hippocampus were placed in 24-well plates (two sections per well), washed three times with 1x TBS (0.1 mol/L Tris/0.15 mol/L NaCl) and 2x SSC (0.3 mol/L NaCl/0.03 mol/L Tri-Na-citrate pH 7). Antigen retrieval was performed with 2x SSC pH=8 by heating to 85° for 30 minutes. Then, sections were washed twice in 2x SSC and three times in 1x TBS pH 7.5, before blocking them for 1.5 hours in 10% fetal bovine serum (Gibco)/0.1% Triton X-100/1x TBS at room temperature (Note: for anti-mouse antibody, another blocking step with M.O.M solutions was performed before this blocking step). After the blocking, the primary antibodies, i.e., rabbit anti-ZO-1, rabbit anti-occludin, rabbit anti-Arg-II, mouse anti-IgG, rat anti-CD31, and goat anti-CD31 diluted in 1% FBS/0.1% Triton X-100/1x TBS, were added to the sections and incubated overnight at 4°C. The next day, sections were washed with 1x TBS and subsequently incubated with the following secondary antibodies: Alexa Fluor 488-conjugated goat anti-mouse IgG (H + L), Alexa Fluor 488-conjugated goat anti-rabbit IgG (H + L), Alexa Fluor 488-conjugated goat anti-rat IgG (H + L) and Alexa Fluor 568-conjugated goat anti-mouse IgG (H + L), or Alexa Fluor 488-conjugated donkey anti-goat IgG (H + L) and Alexa Fluor 594-conjugated goat anti-rabbit IgG (H + L), diluted 1:400 in 1% FBS/0.1% Triton X-100/1x TBS for 3 hours at room temperature. Tissue sections were stained with 300 nmol/L of 4′,6-diamidino-2-phenylindole (DAPI) for 10 minutes. Finally, the tissue sections were washed again twice in 1x TBS and mounted on glass microscope slides. Fluorescent images were taken by using a confocal microscope (Leica TCS SP5). Images were processed with the Leica Application Suite Advanced Fluorescence 2.7.3.9723 according to the study by Schnell et al [27]. Immunostained sections were quantified using ImageJ version 1.49.

For immunostaining of cultured cells, cells were cultured onto glass coverslips, fixed with 4% PFA for 15 minutes at room temperature, and permeabilized with 0.3% Triton X-100, then blocked with 1% BSA + 10% goat serum in 1x PBS for one hour at room temperature. Cells were then incubated with anti-ZO-1 and anti-VE-cadherin as the primary antibodies at 4 °C overnight. After three washes with 1x PBS, cells were incubated for two hours with fluorophore-conjugated secondary antibodies at room temperature. DAPI (4, 6-diamidino-2-phenylindole) was applied to the samples after the final wash to visualize cell nuclei. Finally, the glasses were washed again in 1x PBS and mounted on glass microscope slides. Fluorescent images were taken by using a confocal microscope (Leica TCS SP5).

### Endothelial permeability assay

*In vitro* cell permeability was assessed by trans-well permeability assay. Briefly, the hCMEC/D3 cells were grown to confluency on trans-well polyester membrane inserts (0.4 μm pore size, 6.5 mm diameter; corning 3470), which were first coated with rat collagen-I. Streptavidin-HRP-containing medium (15 μl streptavidin-HRP per 1 ml of serum-free medium) was added to the top chamber. After incubation for 20 minutes, 20 µl of media from the lower chamber was transferred to a new 96-well plate. The paracellular permeability of the hCMEC/D3 cell monolayers to streptavidin-HRP was assessed by adding TMB substrate to the medium from lower chamber in the 96-well plate. The absorbance at 450 nm (OD450) was acquired with a microplate reader as described previously [28].

**Figure 1.**
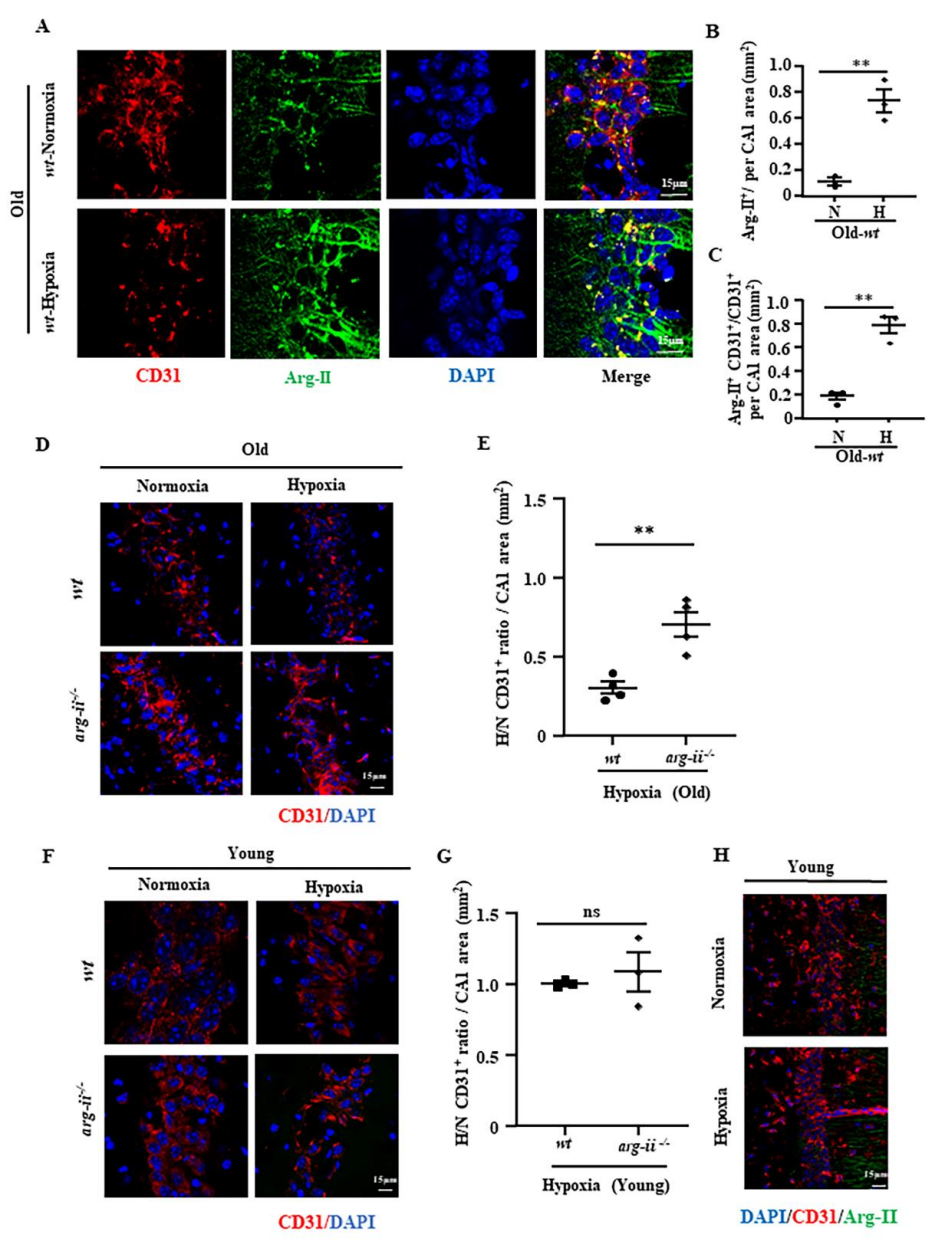
**Hypoxia increases Arg-II in cerebrovascular endothelial cells in old mice**. The coronal hippocampus sections from young (3 to 6 months) and old male mice (20 to 24 months) exposed to normoxia (21% O_2_) or acute hypoxia (8% O_2_) for 24 hours, were subjected to immunostaining for indicated cellular markers followed by counterstaining for nuclei with DAPI. **(A)** Immunostaining of CD31 (red), Arg-II (green), and DAPI (blue) and merged pictures in hippocampal CA1 region from old mice. **(B)** Quantification of total Arg-II signals in the hippocampal CA1 region of old mice under normoxia (N) or hypoxia (H) condition. **(C)** Quantification of endothelial Arg-II as revealed by Arg-II and CD31 co-staining signals in the CA1 region. Endothelial Arg-II was quantified as Arg-II^+^CD31^+^/CD31^+^ ratio. **(D)** Immunostaining of CD31 (red) and DAPI (blue) from old *wt* and *arg-ii^-/-^* mouse hippocampal CA1 regions. **(E)** quantification of CD31 signals from hypoxic (H) vs normoxic (N) mice in CA1 (H/N CD31^+^ ratio per mm^2^ CA1 area). **(F, G)** Immunostaining of CD31 (red) and DAPI (blue) in young mouse hippocampal CA1 regions and quantification of the CD31 signals. The CD31 signals obtained from hypoxic mice were normalized to the corresponding normoxia groups (H/N CD31^+^ ratio per mm^2^ CA1 area). The data were analyzed by two-tailed (B, C, E, G) unpaired t-test with Welch’s correction. n=3 (B, C, G) or 4 (E). **p≤0.01 between the indicated groups. N: normoxia, H: hypoxia. *wt*: wild type, *arg-ii^-/-^*: *arg-ii* gene knockout. Scale bar: 15 µm.

*In vivo* cerebrovascular leakage was assessed by dual immunostaining for CD31 (to label endothelial cells) and blood-protein IgG in hippocampal sections. The relative area of extravascular IgG-positive signal was quantified in each image, employing extravascular IgG leakage as an index of BBB disruption [29].

**Figure 2.**
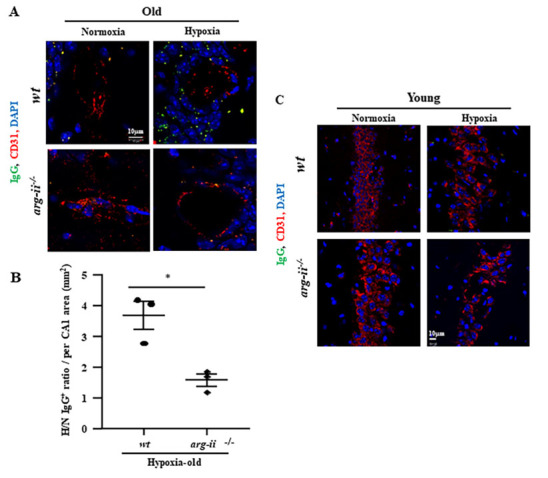
**Arg-II mediates hypoxia-induced BBB leakage in hippocampus of old mice**. The coronal hippocampal sections were prepared as described in Fig. 1. **(A)** Co-immunostaining for IgG (green), CD31 (red), and DAPI (blue) of old mouse hippocampal sections. **(B)** Quantification of extravascular IgG signals from hypoxic (H) vs normoxic (N) mice in CA1 (H/N IgG^+^ ratio per mm^2^ CA1 area). **(C)** Co-immunostaining of IgG (green), CD31 (red), and DAPI (blue) of hippocampal sections from young mice. The data were analyzed by the two-tailed unpaired t-test with Welch’s correction. n=3 (B). *p≤0.05 between the indicated groups. *wt*: wild type, *arg-ii^-/-^*: *arg-ii* gene knockout. Scale bar: 10 µm.

### Statistics

In all experiments, n indicates the number of independent experiments or the number of animals. The Shapiro-Wilk test (that determines normality when n≥3) was used first to determine whether the data deviated from Gaussian distributions. Since all data are normally distributed, parametric statistical analysis was performed. To compare the averages of two groups, the Student's t-test for unpaired observations using Welch’s correction was performed. To analyze the difference between the means of more than two groups, analysis of variance (ANOVA) was performed followed by multiple comparisons with uncorrected Fisher’s least significant difference (LSD) test. All data are expressed as mean ± SEM. Differences were considered statistically significant at two tailed *p*≤0.05. The n indicates the number of individual animals used in each group or of individual experiments when conducted with cells.

**Figure 3.**
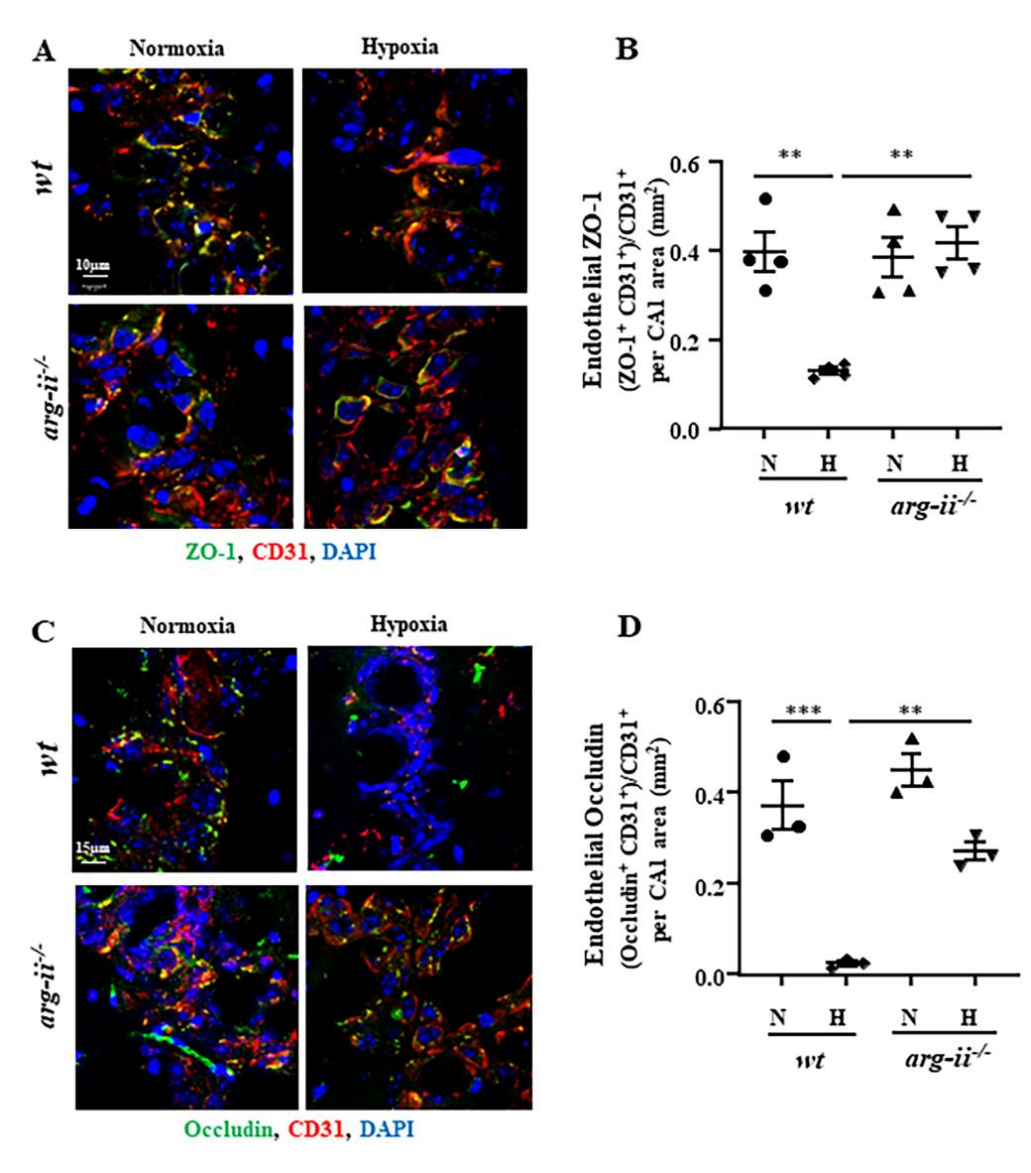
**Arg-II mediates hypoxia-induced decrease in endothelial TJ proteins in hippocampus of old mice**. Coronal sections of hippocampus were prepared as described in Fig. 1. **(A)** Co-immunostaining for ZO-1 (green), CD31 (red), and DAPI (blue). Scale bar: 10 µm. **(C)** Co-immunostaining for occludin (green), CD31 (red), and DAPI (blue). Scale bar: 15 µm. **(B, D)** Quantifications of endothelial ZO-1, i.e., ZO-1^+^CD31^+^ and endothelial occludin, i.e., occludin^+^CD31^+^ areas in relation to total CD31^+^ area in normoxic and hypoxic groups. The data were analyzed by the ordinary one-way ANOVA followed by multiple comparisons with uncorrected Fisher’s LSD test. n=3 animals (D) or 4 (B). **p≤0.01, ***p≤0.001 between the indicated groups. *wt*: wild type, *arg-ii^-/-^*: arg-ii gene knockout. N: normoxia, H: hypoxia.

## RESULTS

### Hypoxia increases Arg-II in brain vascular endothelial cells in aged mice

To study the effect of hypoxia on Arg-II protein level, old male *wt* and *arg-ii*^-/-^ mice (20-24 months in age) were exposed to normoxic or hypoxic (8% O_2_) environments for 24 hours followed by euthanasia and brain collection. Immunostaining of mouse hippocampus sections revealed that hypoxia enhanced Arg-II signals in the CA1 region of the hippocampus (Fig. 1A and 1B). Moreover, vascular endothelial Arg-II signals, as reflected by co-localization with CD31, were significantly increased under hypoxic conditions (Fig. 1A and 1C). This is accompanied by a decrease in CD31 signals (Fig. 1A). Interestingly, the decrease in CD31 signals induced by hypoxia in the old *wt* hippocampal CA1 region was significantly reduced in age-matched *arg-ii^-/-^* mice (Fig. 1D and 1E). In contrast, these effects of hypoxia on endothelial Arg-II and CD31 were not observed in young mice (3 to 6 months) (Fig. 1F to 1H). Of note, Arg-II was also present and induced by hypoxia in CD31 negative cells in the *wt* old mice (Fig. 1A), suggesting that besides endothelial cells, other cell types also express Arg-II in response to hypoxia. These results demonstrate that hypoxia induces Arg-II in the cerebrovascular endothelial cells *in vivo.*

**Figure 4.**
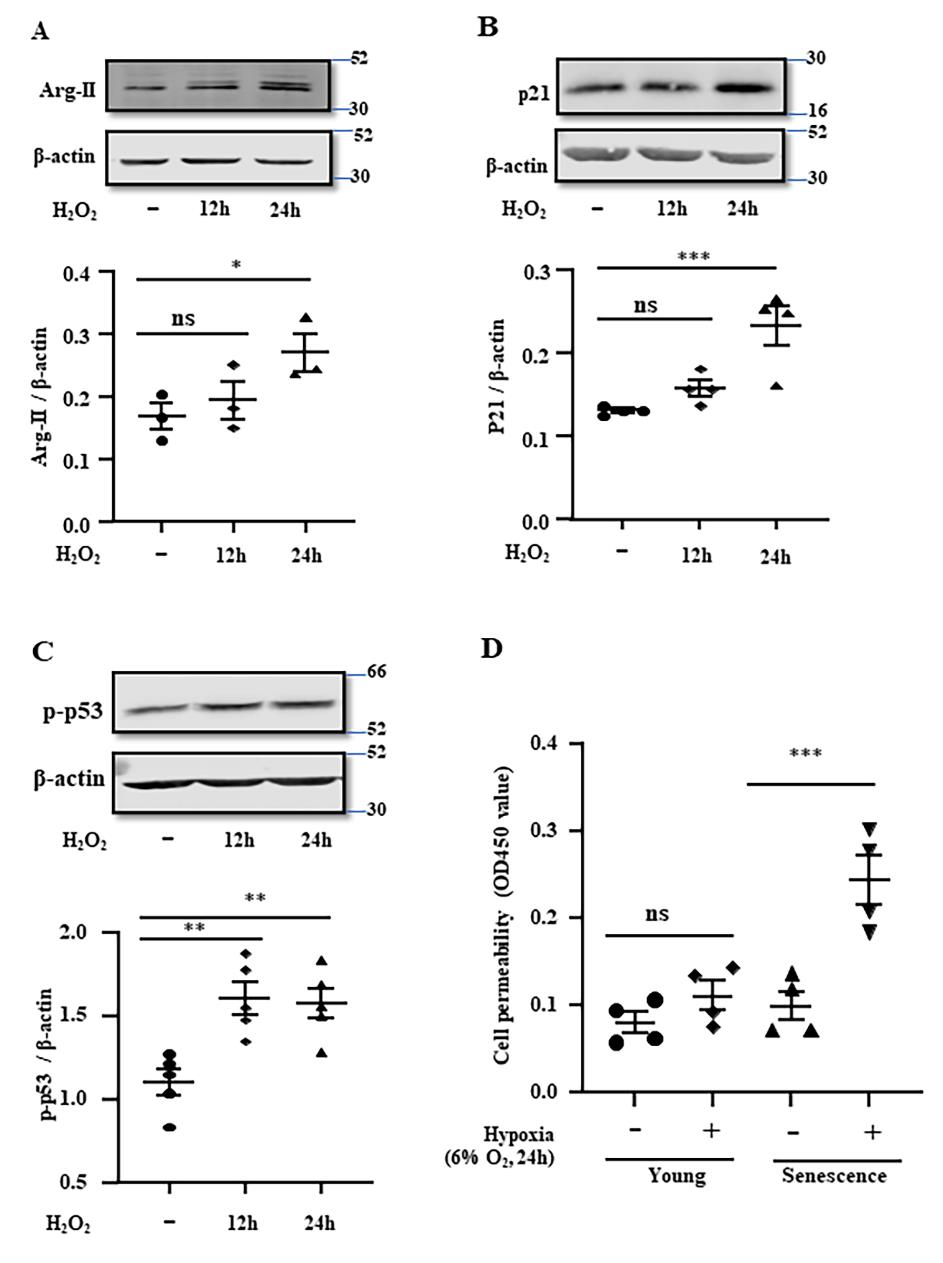
**Senescent human cerebrovascular endothelial cells are more vulnerable to hypoxia**. hCMEC/D3 cells were treated with 100 µmol/L of H_2_O_2_ for 12 and 24 hours and then subjected to immunoblotting analysis of **(A)** Arg-II, **(B)** p21 and **(C)** p53-S15 (p-p53). β-actin was used as the loading control. Quantification of the signals was presented as the ratio of specific protein signal / b-actin in the corresponding graphics. **(D)** hCMEC/D3 were first treated with H_2_O_2_ (100 µmol/L) for 24 hours to induce senescence and then exposed to either normoxia (21% O_2_) or hypoxia (6% O_2_) for 24 hours and then subjected to *in vitro* trans-well endothelial permeability assay. The data were presented as the value measured at OD450 nm. All the data were analyzed by the ordinary one-way ANOVA followed by multiple comparisons with uncorrected Fisher’s LSD test. n=3 (A), 4 (B, D), 5 (C). *p≤0.05, **p≤0.01, ***p≤0.001, ns = not significant between the indicated groups.

### Arg-II mediates hypoxia-induced BBB disruption in hippocampus of aged mice

Next, the role of Arg-II in hypoxia-induced increase in endothelial permeability and BBB disruption was investigated in the mouse model *in vivo*. Co-immunostaining of blood-protein IgG with endothelial marker CD31 revealed a significant increase in extravascular IgG-signals in the hippocampus from the old *wt* mice exposed to hypoxia as compared to the mice under normoxia conditions (Fig. 2A and 2B), demonstrating an enhanced extravascular accumulation of blood-protein IgG, which is indicative of BBB disruption. Remarkably, this effect of hypoxia on BBB leakage was prevented in age-matched *arg-ii^-/-^* mice (Fig. 2A and 2B). In contrast to the old *wt* mice, hypoxia did not induce BBB-disruption as revealed by lack of extravascular IgG accumulation in the young *wt* mice (Fig. 2C).

### Arg-II mediates hypoxia-induced decrease in endothelial TJ proteins in hippocampus of old mice

Further, we investigated whether Arg-II-mediated BBB disruption in old mice under hypoxia condition is attributable to its effect on TJ proteins. Immunostaining of hippocampal coronal sections revealed a strong decrease in endothelial ZO-1 (Fig. 3A and 3B) and occludin (Fig. 3C and 3D) as demonstrated by co-localization with endothelial marker CD31 in old (20-24 months in age) *wt* mice exposed to hypoxia. These effects of hypoxia on endothelial TJ proteins were prevented in *arg-ii*^-/-^ mice (Fig. 3A to 3D). The results demonstrate that Arg-II plays an important role in hypoxia-induced reduction of TJ proteins, leading to enhanced endothelial cell permeability and BBB disruption *in vivo*, particularly in aging.

**Figure 5.**
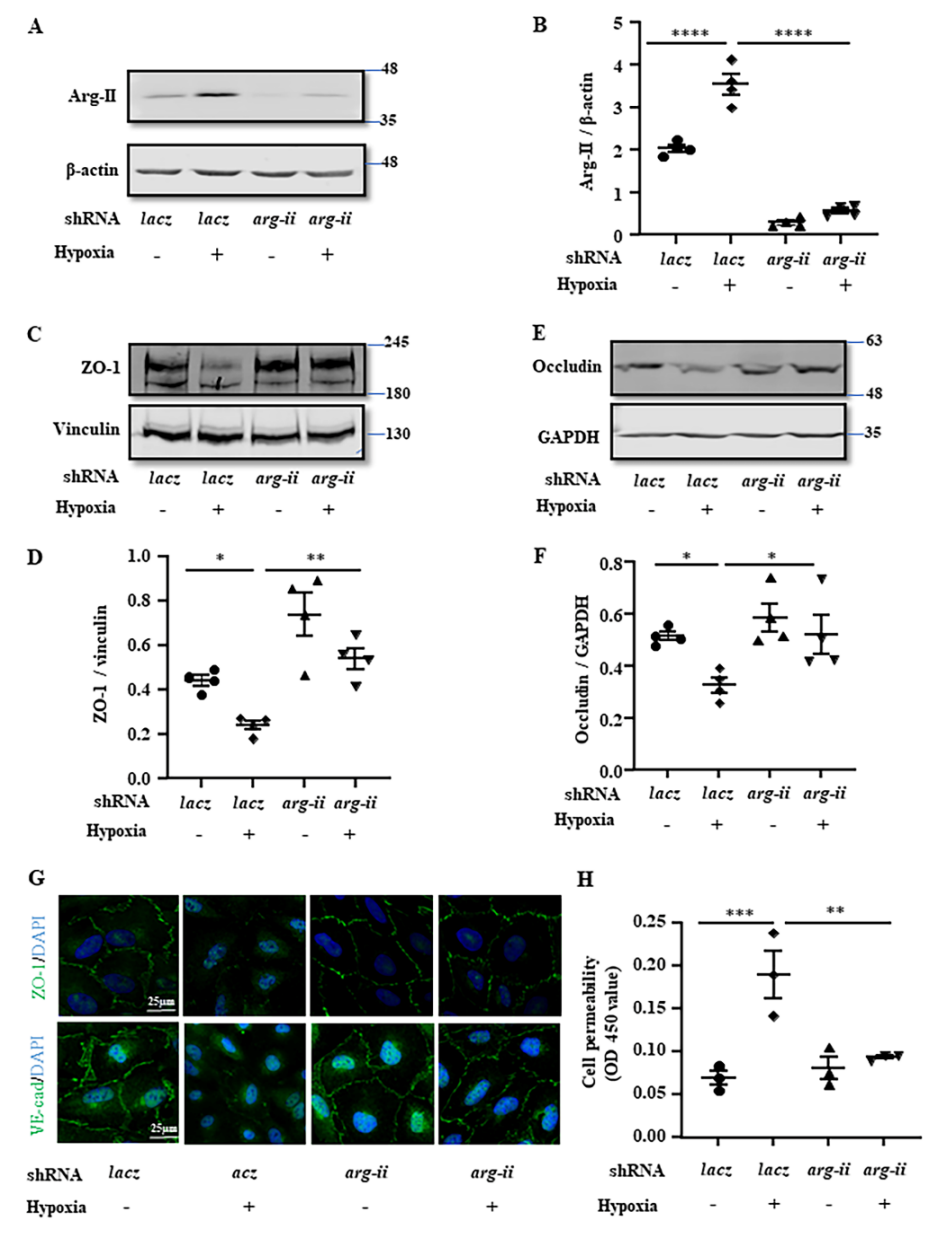
**Arg-II mediates hypoxia-induced reduction of junctional proteins and increase of permeability in cultured cerebral vascular endothelial cells**. hCMEC/D3 cells were transduced with the rAd/U6-*lacz*^shRNA^ as the control or rAd/U6-*arg-ii*^shRNA^ to silence *arg-ii*. 48 hours post transduction, cells were exposed to either normoxia (21% O_2_) or hypoxia (1% O_2_) for 24 hours and then subjected to **(A, C, E)** immunoblotting analysis of Arg-II, ZO-1, and occludin. β-actin, vinculin and GAPDH were used as the loading control. **(B, D, F)** Quantification of the signals was presented as the ratio of specific protein signal / reference protein in the corresponding graphics. **(G)** Immunostaining for ZO-1 (green) or VE-cadherin (green) followed by counterstaining of the nuclei with DAPI (blue). Scale bar: 25 µm. **(H)** *In vitro* trans-well endothelial permeability assay. The data were presented as the value measured at OD450 nm. The data were analyzed by the ordinary one-way ANOVA followed by multiple comparisons with uncorrected Fisher’s LSD test. n=3 (G, H) or 4 (B, D, F). *p≤0.05, **p≤0.01, ***p≤0.001, ****p≤0.0001 between the indicated groups.

**Figure 6.**
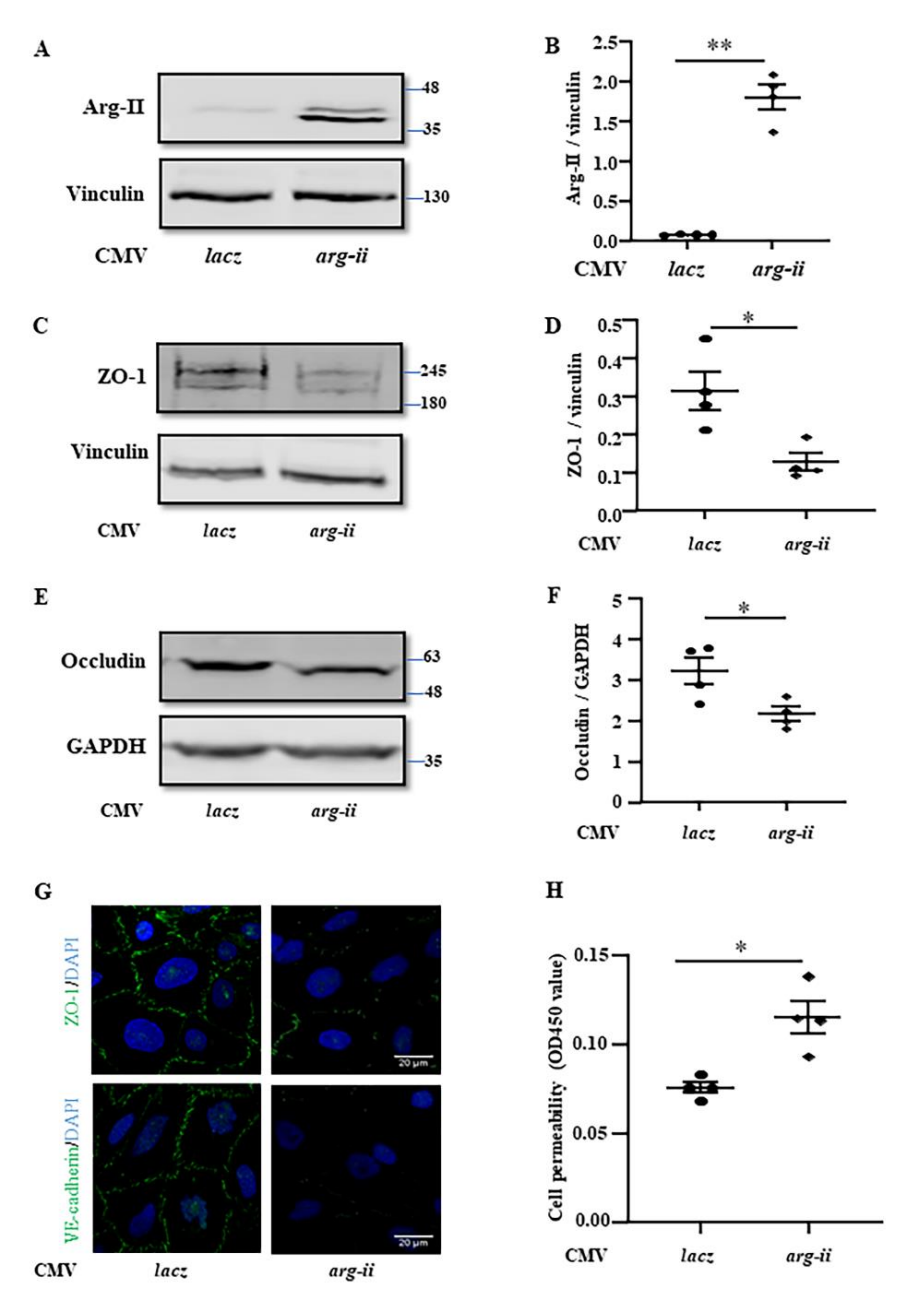
**Overexpression of *arg-ii* decreases junctional proteins and increases permeability in cultured cerebral vascular endothelial cells**. hCMEC/D3 cells were transduced with the rAd/CMV-*lacz* as the control or rAd/CMV-*arg-ii* to overexpress *arg-ii* for 48 hours and then subjected to immunoblotting analysis of Arg-II **(A)**, ZO-1 **(C)** and occludin **(E)**. Vinculin or GAPDH was used as loading controls. **(B, D, F)** Quantification of the signals was presented as the ratio of specific protein signal / reference protein in the corresponding graphics. **(G)** Immunofluorescence staining of ZO-1 (green) or VE-cadherin (green) followed by counterstaining of the nuclei with DAPI (blue). Scale bar: 20 µm. **(H)** *In vitro* trans-well endothelial permeability assay and the data were presented as the value measured at OD450 nm. The data were analyzed by two-tailed (6B, D, F, H) unpaired t-test with Welch’s correction. n=3 (G), 4 (B, D, H, F). *p<0.05, **p≤0.001 between the indicated groups.

### Senescent human cerebrovascular endothelial cells are more vulnerable to hypoxia

Next, we explored the underlying mechanism of Arg-II-mediated decrease in endothelial TJ proteins and increase in endothelial permeability by employing an *in vitro* hCMEC/D3 cell culture model (human brain microvascular endothelial cells). The vulnerability of aging cells to hypoxia-induced endothelial permeability was studied. To do so, senescence of hCMEC/D3 was induced by H_2_O_2_, a widely used agent to induce cellular senescence [30]. Treatment of the cells with 100 µmol/L of H_2_O_2_ for 12 and 24 hours increased Arg-II along with enhanced p21 and p53-S15 reflecting H_2_O_2_-induced cellular senescence (Fig. 4A to 4C). *In vitro* permeability assay revealed that exposure to a relatively mild hypoxia (6% O_2_) for 24 hours markedly increased cell permeability in the senescent cells, while only marginal increase was observed in the non-senescent “young” cells (Fig. 4D), demonstrating that senescent human cerebrovascular endothelial cells are more vulnerable to hypoxia.

It is to mention that more severe hypoxia, i.e., lower than 5% O_2_ also causes increased endothelial permeability in both “young” and senescent cells to a similar degree (data not shown). Therefore, to further investigate the mechanism of Arg-II-mediated decrease in endothelial TJ proteins and increase in endothelial permeability, “young” hCMEC/D3 cells were exposed to a more severe hypoxic condition (1% O_2_) for 24 hours, which enhanced Arg-II protein levels with concomitant decrease in TJ proteins ZO-1 and occludin as compared to the normoxic control group, and all these effects were reversed by *arg-ii* silencing (Fig. 5A to 5F). Moreover, the integrity of ZO-1 and VE-cadherin in the confluent monolayer of endothelial cells was disrupted under hypoxic conditions, which was also prevented by *arg-ii* silencing (Fig. 5G). In accordance, hypoxia-increased endothelial permeability as assessed by the trans-well permeability assay was prevented by *arg-ii* silencing (Fig. 5H).

Conversely, overexpression of *arg-ii* (Fig. 6A and 6B) decreased levels of ZO-1 and occludin in endothelial cells (Fig. 6C to 6F). The integrity of ZO-1 and VE-cadherin of the confluent monolayer of endothelial cells was disrupted by *arg-ii* overexpression (Fig. 6G). Accordingly, the endothelial monolayer permeability was enhanced by *arg-ii* overexpression (Fig. 6H).

These results with hCMEC/D3 cell culture model further confirm the *in vivo* observation that Arg-II plays a critical role in the hypoxia-induced disruption of endothelial integrity with downregulation and/or disorganization of endothelial TJ and AJ proteins.

**Figure 7.**
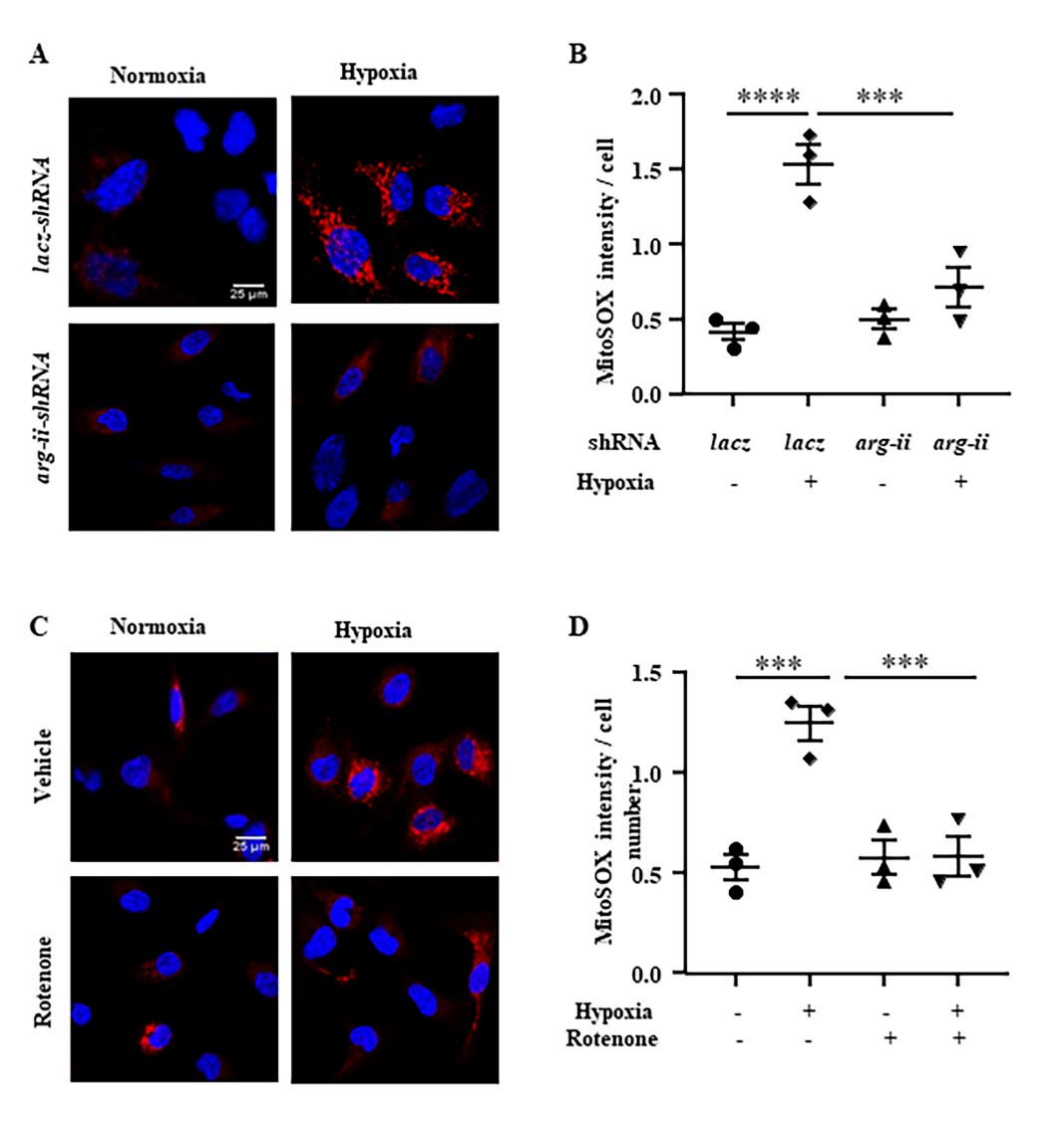
**Hypoxia promotes mtROS production through Arg-II. (A)** hCMEC/D3 were prepared as described in Fig. 5. After exposure to normoxia (21% O_2_) or hypoxia (1% O_2_) for 24 hours, cells were stained with MitoSOX for detection of mtROS (red) followed by nuclei staining with Hoechst 33342 (blue). Scale bar: 25 µm. **(C)** Cells were pre-treated with rotenone (2 μmol/L) for one hour and subsequently exposed to hypoxia (1% O_2_) for 24 hours. Cells were then subjected to MitoSOX staining (red) followed by nuclei staining with Hoechst 33342 (blue). Scale bar: 25 µm. **(B, D)** Quantifications of the MitoSOX intensity / cell. The data were analyzed by the ordinary one-way ANOVA followed by multiple comparisons with uncorrected Fisher’s LSD test. n=3 (B, D). ***p≤0.001 and ****p≤0.0001 between the indicated groups.

**Figure 8.**
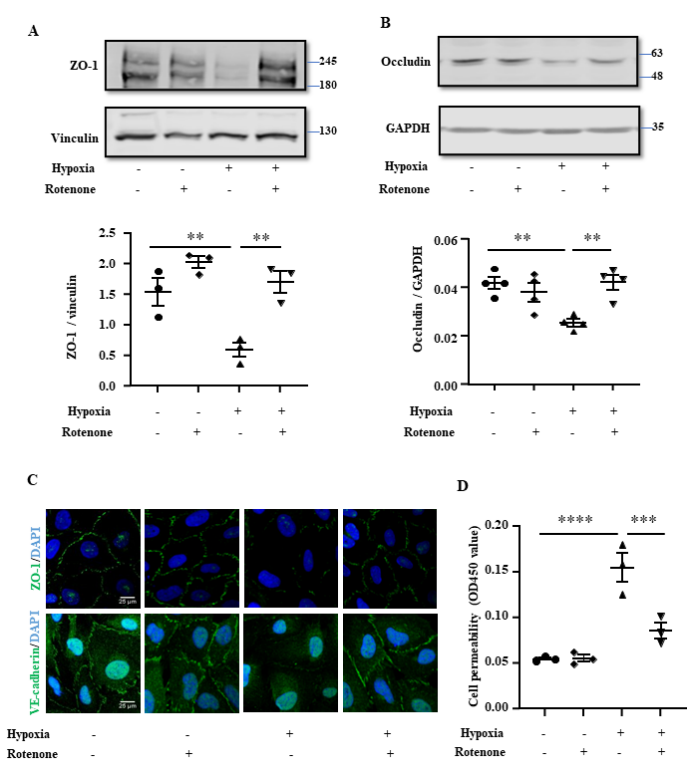
**Hypoxia decreases junctional proteins and increases endothelial permeability via mtROS**. Cells were treated as described in Fig. 7C and then subjected to immunoblotting analysis of ZO-1 **(A)** and occludin **(B)**. Vinculin or GAPDH were used as loading controls. Quantification of the ZO-1/Vincullin or Occludin/GAPDH ratio was presented in the corresponding graphics. **(C)** Immunofluorescence staining of ZO-1 (green) or VE-cadherin (green) followed by counterstaining of the nuclei with DAPI (blue). Scale bar: 25 µm. **(D)** *In vitro* trans-well endothelial permeability assay and the data were presented as the value measured at OD450 nm. The data were analyzed by the ordinary one-way ANOVA followed by multiple comparisons with uncorrected Fisher’s LSD test. n=3 (A, C, D) or 4 (B). **p≤0.01, ***p≤0.001, ****p≤0.0001 between the indicated groups.

### Role of mtROS in hypoxia/Arg-II-induced increase in endothelial permeability

Given that elevated Arg-II promotes hypoxia-induced endothelial ROS production [22], the role of mtROS in the disruption of endothelial integrity under hypoxic conditions was investigated. MitoSOX staining revealed that hypoxia indeed enhanced mtROS production in hCMEC/D3, which was prevented by silencing *arg-ii* in the endothelial cells (Fig. 7A and 7B). Rotenone (2 μmol/L), an inhibitor of mitochondrial respiration complex-I, prevented the increase in mtROS production in the endothelial cells under hypoxic conditions (Fig. 7C and 7D). These results demonstrate that hypoxia enhances mtROS production via Arg-II in the hCMEC/D3 cells.

**Figure 9.**
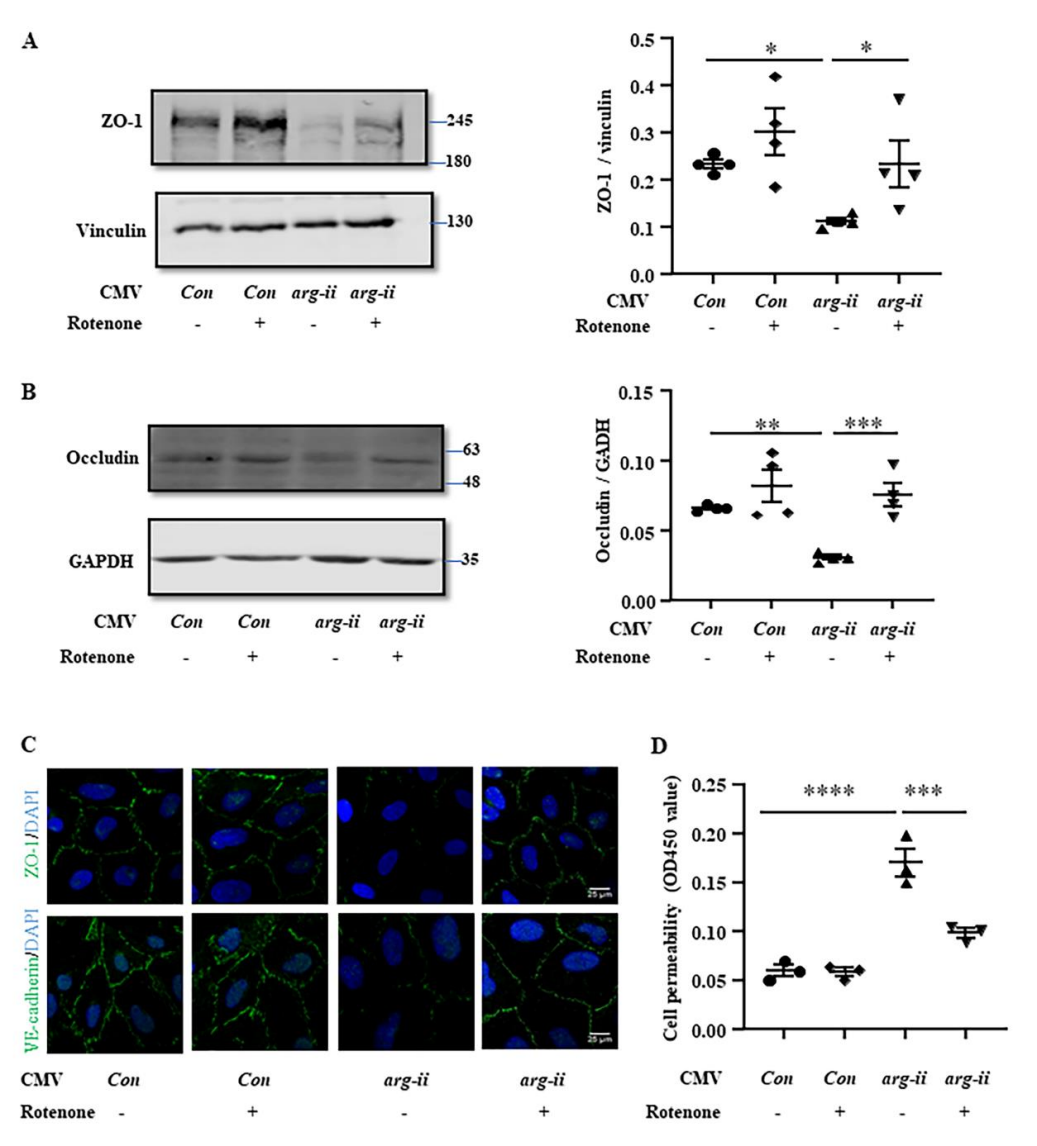
**mtROS mediates Arg-II-induced reduction of junctional proteins and increase in permeability**. The endothelial cells were prepared as described in Fig. 6, except that the cells were pre-treated with rotenone (2 μmol/L) for one hour prior to transduction. **(A and B)** and subjected to immunoblotting analysis of ZO-1 and occludin. Vinculin or GAPDH were used as loading controls. Quantifications of the ZO-1/Vincullin or Occludin/GAPDH was presented in the corresponding graphics. **(C)** Immunofluorescence staining of ZO-1 (green) or VE-cadherin (green) with counterstaining of the nuclei with DAPI (blue). Scale bar: 25 µm. **(D)** *In vitro* trans-well endothelial permeability assay and the data were presented as the value measured at OD450 nm. The data were analyzed by the ordinary one-way ANOVA followed by multiple comparisons with uncorrected Fisher’s LSD test. n=3 (C, D) or 4 (A, B). *p<0.05, **p≤0.01, ***p≤0.001, ****p≤0.0001 between the indicated groups.

In line with these observations, rotenone prevented the hypoxia-induced decrease in ZO-1 and occludin in the endothelial cells (Fig. 8A and 8B). Moreover, the hypoxia-induced disruption of ZO-1 and VE-cadherin integrity of the endothelial monolayer was prevented by rotenone (Fig. 8C). Accordingly, the hypoxia-induced increase in endothelial permeability was also prevented by rotenone (Fig. 8D). Furthermore, the decreased endothelial ZO-1 and occludin levels (Fig. 9A and 9B), disruption of ZO-1 and VE-cadherin integrity of the endothelial monolayer (Fig. 9C), as well as the enhanced endothelial permeability caused by Arg-II overexpression (Fig. 9D) were all reduced or prevented by rotenone. These results demonstrate that Arg-II contributes to hypoxia-induced decrease and disorganization of TJ and AJ proteins, leading to enhanced endothelial leakage through mtROS.

## DISCUSSION

Previous studies show that hypoxia is a potent stimulus for Arg-II protein elevation in endothelial cells [22, 31-34]. In this work, we extend these findings to the human brain microvascular endothelial cells *in vitro* and in aged mice *in vivo.* The results demonstrate an enhanced vulnerability of endothelial permeability and BBB dysfunction in response to hypoxia in senescent endothelial cells and in aged mice as compared to non-senescent cells and young animals, respectively. Moreover, we provide evidence that Arg-II mediates hypoxia-induced endothelial hyperpermeability and reduction of protein levels of TJ and AJ proteins such as ZO-1, occludin, and VE-cadherin. These effects are exerted through mtROS generation caused by Arg-II.

While the overall role of Arg-II has remained largely unknown for some time, more recent studies have begun to uncover its role in vascular endothelial damage and aging via oxidative stress [19] and mitochondrial dysfunction [35]. Identified as the predominant isozyme in human and mouse endothelial cells, Arg-II is implicated in the regulation of endothelial senescence [19, 36] as well as dysfunctions in many disease status and aging [19, 24, 37, 38]. Despite these recognitions, the role and mechanisms of Arg-II function are still poorly understood, particularly in the neurological or cerebral vascular (dys)function and aging. In this study, we investigated the contribution of Arg-II to hypoxia-induced BBB disruption. In line with previous reports in other cell types and in endothelial cells from other regions [22, 39], we show for the first time that Arg-II is upregulated by hypoxia in cerebral vascular endothelial cells both *in vitro* in cultured hCMEC/D3 cells, a frequently used *in vitro* model of human BBB [40] as well as *in vivo* in mouse hippocampal blood vessels. Of note, the hypoxia-induced Arg-II level as well as the detrimental effects of acute hypoxia on BBB are mainly observed in old but not young mice under 8% hypoxia. This age-associated vulnerability of BBB to hypoxia is supported by a recently published study from another group [16]. Furthermore, we demonstrate a decrease and/or disorganization of the TJ and AJ proteins in hippocampal CA1, a highly vascularized region in response to hypoxia, which is caused by elevated Arg-II levels. This conclusion is supported by the fact that overexpression of Arg-II *in vitro* in hCMEC/D3 cells decreases endothelial cell-cell junctional protein levels and disorganization of ZO-1, occludin, and VE-cadherin, and increases endothelial permeability. Conversely, silencing or ablation of Arg-II prevents the hypoxia-induced decrease in TJ and AJ proteins and the increase in endothelial permeability both *in vitro* in cell culture model and *in vivo* in old mice. Similar findings are reported by another study showing that the continuity of ZO-1 expression is disrupted during 24 hours of hypoxia, which correlates with a decrease of the protein levels to more than 30% [41].

ROS are generated at sites of inflammation and injury, and at low levels, they function as signaling intermediates in the regulation of fundamental cell activities such as growth and adaptation responses [42]. At higher concentrations, however, ROS can cause cell injury and death and endothelial dysfunction [42]. The vascular endothelium which regulates the passage of macromolecules and circulating cells from blood to tissue, is a major target of oxidative stress in vascular disease [43]. It is well known that hypoxia causes mitochondrial dysfunction, enhances mtROS generation, and leads to cellular damage, e.g., in renal diseases [44]. Compared to other organs or tissues, the brain is especially vulnerable to oxidative stress-induced damage, which is due to its high rate of oxygen consumption, high polyunsaturated lipid content, and the relative paucity of classic antioxidant enzymes [45]. Our previous studies demonstrate a role of Arg-II in promoting mitochondrial dysfunction and ROS production in aging and hypoxia in endothelial cells and in other cell types [19]. In the current study we show that Arg-II mediates the hypoxia-induced decrease in junctional proteins, as well as the increase in permeability through mtROS. Indeed, inhibition of mtROS prevents Arg-II-induced increase in endothelial permeability and the decrease or disorganization of TJ and AJ proteins, which is more pronounced in senescent cells and aged mice. It is not that surprising that elevated Arg-II levels cause mitochondrial oxidative stress or dysfunction, since Arg-II is a mitochondrial enzyme and a dysregulated homeostasis of Arg-II expression is involved in mitochondrial dysfunction, participating in pathophysiology of diseases. The question of how Arg-II enhances mtROS levels remains unanswered. This aspect warrants further investigation.

In line with the reports from chronic hypoxia experiments (8%O_2_, 14 days) in aged mouse [16], our study with acute hypoxia (8%O_2_, 24 hours) also shows age-associated BBB leakage, which could be prevented by *arg-ii* knockout. Several studies have reported that BBB function declines with age in rodents and humans [14, 15, 46], which is thought to contribute to age-associated acceleration of neurodegenerative diseases [47]. The underlying mechanisms of age-associated BBB dysfunction are complex and poorly understood. Many mechanisms such as endothelial dysfunction, loss of pericytes function and pericytes-endothelial interaction, dysregulation of reactive astrocytes and microglia cells, as well as diminished neurovascular coupling all play a role in age-associated vulnerability of BBB disruption in response to stressors [47]. The fact that ablation of *arg-ii* prevents hypoxia-induced decrease or disorganization of junctional proteins and BBB disruption implies a critical role of Arg-II in hypoxia-induced BBB disruption particularly in aging. Of note, Arg-II is also upregulated in cell types other than vascular endothelial cells as shown in Fig. 1A and 1H. It remains to be investigated which cell types in the brain, besides endothelial cells, express Arg-II under hypoxia, and what is the functional role of Arg-II in these cell types. Our study provides a novel insight in understanding the pathogenesis of BBB dysfunction under hypoxia and has strong clinical implications, since hypoxia and BBB disruption occur in a broad spectrum of diseases, including ischemic stroke, brain trauma, brain edema, high altitude mountain sickness, pulmonary disorders, cardiovascular diseases, obstructive sleep apnea, and neurodegenerative diseases like Alzheimer's disease and Parkinson's disease. Future work shall investigate whether Arg-II is upregulated under these disease conditions and whether ablation or inhibition of Arg-II could preserve BBB integrity and prevent or limit neurological dysfunction such as impairment of cognitive and motor functions under the disease conditions.

In summary, our current study demonstrates an essential and causal role of Arg-II in the enhancement of endothelial permeability and BBB disruption under hypoxic conditions. This effect is related to decrease or disorganization of endothelial cell-cell junctional proteins, which is mediated by elevated production of mtROS. Thus, targeting the Arg-II-mtROS cascade could be a promising therapeutic strategy to prevent hypoxia-induced brain vascular damage or hypoxia-related neuronal damage, particularly in aging and beyond as discussed above.

## Data Availability

Data supporting the present study are available from the corresponding author upon reasonable request.
